# Anti-Trypanosomal Bufadienolides from the Oocytes of the Toad *Rhinella alata* (Anura, Bufonidae)

**DOI:** 10.3390/molecules29010196

**Published:** 2023-12-29

**Authors:** Candelario Rodriguez, Roberto Ibáñez, Dionisio A. Olmedo, Michelle Ng, Carmenza Spadafora, Armando A. Durant-Archibold, Marcelino Gutiérrez

**Affiliations:** 1 Centro de Biodiversidad y Descubrimiento de Drogas, Instituto de Investigaciones Científicas y Servicios de Alta Tecnología (INDICASAT-AIP), Panamá 0843-01103, Panama; crodriguez@indicasat.org.pa (C.R.); adurant@indicasat.org.pa (A.A.D.-A.); 2 Department of Biotechnology, Acharya Nagarjuna University, Nagarjuna Nagar, Guntur 522510, India; 3 Smithsonian Tropical Research Institute, Balboa, Ancon 0843-03092, Panama; ibanezr@si.edu; 4 Departamento de Zoología, Facultad de Ciencias Naturales, Exactas y Tecnología, Universidad de Panamá, Panamá 0824-03366, Panama; 5 Centro de Investigaciones Farmacognósticas de la Flora Panameña (CIFLORPAN), Facultad de Farmacia, Universidad de Panamá, Panamá 0824-03366, Panama; dolmedo_agudo@hotmail.com; 6 Centro de Biología Celular y Molecular de Enfermedades, INDICASAT AIP, Panamá 0843-01103, Panama; michelle.ng.w@gmail.com (M.N.); cspadafora@indicasat.org.pa (C.S.); 7 Departamento de Bioquímica, Facultad de Ciencias Naturales, Exactas y Tecnología, Universidad de Panamá, Panamá 0824-03366, Panama

**Keywords:** *Rhinella alata*, toads, Bufonidae, oocytes, bufadienolides, anti-trypanosomal activity, *Trypanosoma cruzi*

## Abstract

Amphibians are widely known as a prolific source of bioactive metabolites. In this work, we isolated and characterized compounds with antiparasitic activity from the oocytes of the toad *Rhinella alata* collected in Panama. Bio-guided isolation and structural elucidation were carried out using chromatographic and spectroscopic techniques, respectively. The organic extract was subjected to solid phase extraction followed by HPLC purification of the fraction with in vitro activity against *Trypanosoma cruzi* trypomastigotes. Seven steroids (**1**–**7**) of the bufadienolide family were isolated, and their structures were determined using NMR and MS analyses; of these 19-formyl-dyscinobufotalin, (**3**) is reported as a new natural product. Compounds **1** and **3**–**7** resulted in a good anti-trypanosomal activity profile. Among these, 16β-hydroxyl-hellebrigenin (**1**) and bufalin (**7**) showed significant selectivity values of >5 and 2.69, respectively, while the positive control benznidazole showed a selectivity of 18.81. Furthermore, molecular docking analysis showed compounds **1**, **3** and **7** interact through H-bonds with the amino acid residues GLN-19, ASP-158, HIS-159 and TRP-177 from cruzipain at the catalytic site. Given the lack of therapeutic options to treat American trypanosomiasis, this work can serve as the basis for further studies that aim for the development of bufadienolides or their derivatives as drugs against Chagas disease.

## 1. Introduction

Chagas disease, or American trypanosomiasis, is a zoonotic disease caused by the parasite *Trypanosoma cruzi*. This protozoan is transmitted to humans mainly through the feces of insects of the family Reduviidae [1]. Although Chagas disease is recognized as endemic in Latin America, it has become a worldwide issue, with 10,000 deaths per year and eight million people at risk of infection [2]. There are no specific symptoms for diagnostics during its acute phase; however, once the disease advances, chronic pathological features such as cardiomyopathy, megacolon, megaesophagus and stroke are developed [3]. The low effectiveness and side effects of the available drugs used for the treatment of this disease during its chronic phase highlight the need for more efficacious and safer anti-trypanosomal treatments [4].

Bufadienolides are polyhydroxy steroids that contain an unsaturated 2H-pyran-2-one ring at the β-17 position [5]. Because of their wide bioactivity against human cancer cell lines and their great chemical diversity, bufadienolides have attracted considerable attention in recent years [6]. These metabolites are widely distributed in nature and have been reported in fireflies, mammals, plants, snakes and toads [7]. Bioprospecting research has revealed that bufadienolides, isolated from some amphibian species, have promising antibiotic and antiparasitic properties [8].

Herein, we report the bio-guided isolation of anti-trypanosomal bufadienolides from the oocytes of the toad *Rhinella alata* (Thominot, 1884) from Panama [9]. Seven compounds were isolated, and their chemical structures were elucidated by spectroscopic techniques. Compounds were tested against trypomastigotes of *T. cruzi* and evaluated on Vero cells for mammalian cytotoxicity. Furthermore, bufadienolides **1**, **3** and **7** were analyzed by molecular docking with cruzipain, revealing chemical interactions with amino acid residues at the active site.

## 2. Results and Discussion

### 2.1. Isolation and Structural Elucidation of Bufadienolides

**Figure 1:**
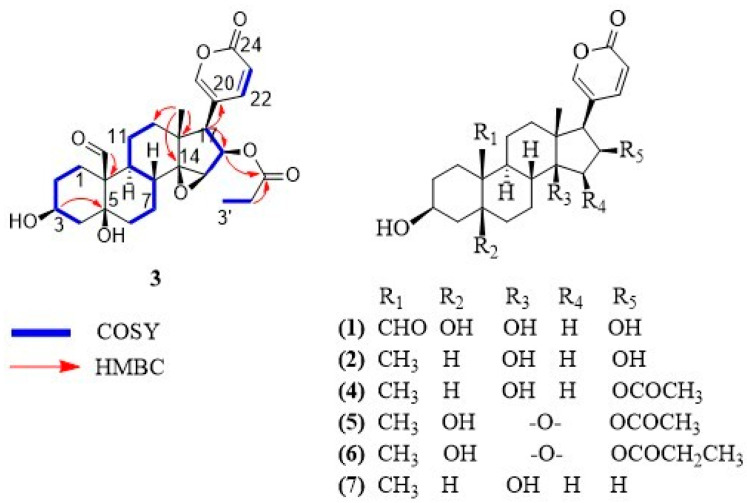
Chemical structures of bufadienolides **1–7**.

The crude extract from the oocytes of *R. alata* was prepared by maceration with a mixture of methanol and chloroform and then submitted to C-18 SPE purification. The fraction eluted at 80% CH_3_OH showed anti-trypanosomal activity and was further analyzed by semi-preparative RP-HPLC (Figure S1). Compounds **1**–**7** were isolated, and their structures, depicted in Figure 1, were determined based on extensive spectroscopic analysis and comparison of their data with those reported in the literature. While compounds **1**, **2** and **4**–**7** corresponded to known compounds, bufadienolide **3** is reported as a new natural product.

Compound **3** was isolated as a white solid. The molecular formula was determined as C_27_H_34_O_8_ based on its HR-MALDI-MS data, which showed the molecular ion at *m/z* 487.2283 [M + H]^+^ (calculated for C_27_H_35_O_8_, 487.2326). Eleven degrees of unsaturation were inferred from the molecular formula. The ^1^H-NMR and ^13^C-NMR spectra of compound **3** show the feature signals for a 2H-pyran-2-one moiety [δ_H_ 6.24 (1H, dd, *J* = 0.8, 9.7 Hz), 7.36 (1H, m), 8.02 (1H, m); δ_C_ 114.2, 118.2, 150.9, 153.6, 164.0], a tertiary methyl [δ_H_ 0.81 (3H, s); δ_C_ 17.3], a secondary methyl [δ_H_ 0.94 (3H, t, *J* = 7.8 Hz); δ_C_ 9.3], three oxygenated methines [(δ_H_ 3.75, m; δ_C_ 60.7), (δ_H_ 4.15, m; δ_C_ 68.0), (δ_H_ 5.47, m; δ_C_ 76.3)], two oxygenated quaternary carbons [δ_C_ 75.8, 73.1] and two carbonyl carbons characteristic of aldehyde and ester [δ_C_ 210.1, 174.9]. Comparison of the NMR signals (Table 1) of compound **3** with those of the reported bufadienolide dyscinobufotalin [10] reveal the data of the steroidal nucleus of the two compounds are very similar, indicating that compound **3** has the same bufadienolide core with two carbinols at C-3 and C-5, an epoxy group at C-14 and C-15 and the esterification of the hydroxyl group at C-16 with propionic acid. The main difference consists of the presence of an aldehyde group at C-19 in compound **3** instead of the angular methyl group at C-19 of dyscinobufotalin [10]. HMBC correlations between the aldehyde proton at δ_H_ 10.00 and the quaternary carbon at C-10 allowed for the assignment of the aldehyde group at C-19 (Figure 1). On the basis of spectral data, compound **3** was identified as 19-formyl-dyscinobufotalin.

Furthermore, six steroids of the bufadienolide family were identified as 16β-hydroxyl-hellebrigenin (**1**) [11], desacetyl-bufotalin (**2**) [5], bufotalin (**4**) [5], cinobufotalin (**5**) [12], dyscinobufotalin (**6**) [10] and bufalin (**7**) [5].

### 2.2. Anti-Trypanosomal Activity and Mammalian Cytotoxicity

The trypanosomaticidal activity of bufadienolides **1**–**7** was evaluated in vitro against *T. cruzi*. The ability of these compounds to inhibit the growth of the trypomastigote form was evaluated at four different concentrations. The results show that bufadienolides **1**, **3**, **4**, **5**, **6** and **7** reduce the growth of *T. cruzi* while compound **2** is inactive at the screening concentration of 10 µg/mL. Bufadienolides **3** and **7** present EC_50_ values (0.19 and 3.93 µM, respectively), compound **3** being even more active than the positive control benznidazole (Table 2). The cytotoxicity of bufadienolides against mammalian cells was also tested in vitro using healthy epithelial kidney Vero cells. The IC_50_ values of bufadienolides **1–7** range from 0.02 to >135 µM. As shown below, compounds **1**, **2** and **4** present the lowest cytotoxic effects. Both the antiparasitic and cytotoxic activities exhibited by each bufadienolide assessed are shown in Table 2. Selectivity was analyzed by the cytotoxicity on Vero cells/anti-trypanosomal activity ratio.

### 2.3. Molecular Docking of Bufadienolides **1**, **3** and **7**

**Figure 2:**
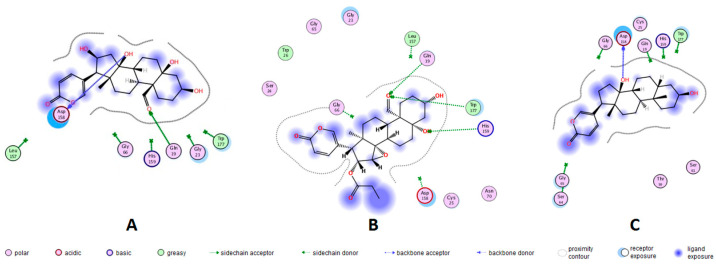
Representation in 2D format for molecular docking of bufadienolides **1**, **3** and **7** with active site amino acid residues of cruzipains. X-ray crystallographic structures of the proteins were extracted from the Protein Data Bank (PDB). Bufadienolide/cruzipain PDB ID = (**A**) 16β-hydroxyl-hellebrigenin (**1**)/1EWO; (**B**) 19-formyl-dyscinobufotalin (**3**)/1EWO; and (**C**) bufalin (**7**)/2AIM.

A docking protocol was validated, and its reproducibility was confirmed by the root-mean-square deviation (RMSD) values. The RMSD (in Å) for each cruzipain-co-crystallized inhibitor complex are as follows: 2.3586 for 1EWO-VSC, 1.9060 for 2AIM-ZRA, 1.6817 for 1U9Q-186 and 2.9319 for 4XUI-2VC. In addition, relative docking scores for each complex resulted in −7.0484, −6.9388, −8.2353 and −8.1351. After validation of the protocol accuracy, interactions of anti-trypanosomal bufadienolides **1**, **3** and **7** with the active site of proteins were studied via docking calculations. Table 3 shows the calculated parameters for the best pose obtained for each bufadienolide tested. The energy variation for the predictions obtained by simulation ranged from −2.2 to +1.3 kcal/mol. The isolated bufadienolides **1**, **3** and **7** displayed interactions with cruzipain. Bufadienolide **1** shows H-bonds with ASP-158 and GLN-19 at a distance of 2.10 Å and 2.32 Å, respectively. On the other hand, bufadienolide **3** presents three H-bonds with amino acid residues GLN-19, HIS-159 and TRP-177. Bufadienolide **7** formed one H-bond with ASP-158 at a distance of 1.95 Å (Figure 2).

Currently, there is no effective chemotherapeutic treatment available for American trypanosomiasis [4]. Poisonous terrestrial vertebrates are rarely considered for natural product research programs; however, it has been reported that bioactive materials derived from animals have resulted in effective medicines, highlighting the potential of vertebrate animals’ toxins in pharmacology. For instance, teprotide, a nonapeptide isolated from the venom of the snake *Bothrops jararaca*, has served as a template to produce captopril and other angiotensin-converted enzyme inhibitors that currently are routinely medicated for hypertension patients [13]. The alkaloid epibatidine obtained from the skin of the dendrobatid frog *Epipedobates tricolor* presents analgesic properties 200 times more potent than morphine. Epibatidine has served as the template for producing anti-nociceptive derivatives such as tebanicline (ABT-594) that reached clinical phase II [14,15].

In this study, through the analysis of the spectroscopic data, we elucidated the chemical structure of the new bufadienolide **3** from the oocytes of *R. alata*. Moreover, five bufadienolides (**2**, **4**–**7**) were previously reported in amphibians and one in plants (**1**). Bufadienolide **2** was isolated from the poison of the toad *Duttaphrynus melanostictus* as a minor constituent [5]. Compounds **4** and **5** have been obtained from skin and parotoid gland secretions of bufonids but not in toads of the genus *Rhinella* [16]. Bufadienolide **6** was isolated from Chan Su, a traditional Chinese remedy prepared with the poison of Asian toads [10]. Bufadienolide **7** has been identified in bufonid extracts, including oocytes [17].

Due to the marked inhibitory activity against the pump (Na^+^-K^+^)ATPase, the antiparasitic potential of bufadienolides against human pathogens has currently received some attention [18]. In this study, bufadienolides **1**–**7** were evaluated in vitro for inhibition of the growth of the trypomastigote form of *T. cruzi*. According to our data, the substitution of methyl at C-19 by the formyl group increases the anti-trypanosomal activity, as seen in bufadienolide **3** in comparison with bufadienolide **6**. Similarly, in a previous study, hellebrigenin showed trypanocidal activity at 91.7 µg/mL, while telocinobufagin, which contains a C-19 methyl group instead, was inactive [19]. Likewise, comparing the activities of bufadienolides **2** and **7**, it can be inferred that the lack of a hydroxyl group at C-16 is required for the anti-trypanosomal activity; however, if this hydroxyl group is esterified, moderate bioactivity is observed (compounds **4**–**6**, Table 2).

Also, previous evaluations on cancer cell lines with bufadienolides revealed a decrease in growth inhibition by the introduction of a hydroxyl group at the C-16 position [20]. Bufadienolide **7** presents higher anti-trypanosomal activity than its 19-OH analog (IC_50_ = 19.4 µM) [21]. On the basis of these results, we can suggest that the presence of a formyl group at C-19 and the esterification of the hydroxyl group at C-16 are essential elements required for the effective biomolecular interactions with components present in the trypomastigote form of *T. cruzi*, which led to the anti-protozoal activity observed.

In mammals, bufadienolides block the transport activity of the (Na^+^-K^+^)ATPase pump by binding to the α-subunit. This inhibition provokes intracellular sodium concentration to increase until cells become depolarized [22]. In *T. cruzi*, there is no report on the identification of this protein; maintenance of the ionic steady-state is carried out by K^+^ channels, Na^+^ efflux pumps and (H^+^)ATPases [23]. Kyoichi, I. et al. [24] cloned and characterized a (Na^+^)ATPase from a gene encoding of *T. cruzi*. After biochemical evaluations it was evidenced that the enzymatic activity of this pump was not inhibited by the steroid (cardenolide) ouabain. In the search for anti-trypanosomal drugs, the most considered targets for Chagas disease are cruzipain, sterol-14α-demethylase, trans-syalidase, trypanothione reductase and tubulin [4,24]. Currently, the mechanism by which bufadienolides cause inhibition of *T. cruzi* is unknown, despite the previous findings suggesting that bufadienolides inhibit the growth of *T. cruzi* parasites by a biochemical pathway other than the blockage of the (Na^+^-K^+^)ATPase pump. In this study, in silico analysis by molecular docking was carried out, using the *T. cruzi* protease cruzipain. Redocking with previously reported co-crystallized inhibitors was employed for protocol validation and active site prediction (Figure S9). Molecular docking with the anti-trypanosomal bufadienolides **1**, **3** and **7** revealed strong interactions with cruzipains. These interactions correspond to H-bonds between amino acid residues GLN-19, ASP-158, HIS-159 and TRP-177 with hydroxyl groups at the C-5 and C-14 positions and formyl at the C-19 position of bufadienolides. The most potent anti-trypanosomal bufadienolide (**3**) presented three interactions with cruzipain, while bufadienolides **1** and **7** showed two and one H-bonds, respectively. Bufadienolide **7** interacts with cruzipain by the hydroxyl at C-14, a functional group essential for bioactivity [25]. No interaction with polar groups at C-16, such as hydroxyl and propionyloxy, was observed for bufadienolides **1** and **3**. Cruzipain contains, at the active site, a catalytic triad formed by GLY-23, CYS-25 and GLY-65 [26]. Furthermore, some amino acid residues have been revealed to be involved in the enzymatic catalysis of cruzipain, such as residues GLN-19, GLY-66, ASP-158, HIS-159 and TRP-177 that keep peptidyl inhibitors anchored to the active site [27]. A molecular docking analysis with 173 compounds reported to be inhibitors of cruzipain showed that in addition to the catalytic triad, the amino acid residues GLN-19 and ASP-158 are involved in ligand interactions with a frequency of 47% and 44%, respectively [26].

Selectivity is a well-accepted parameter of the safety and pharmacological activity of a compound [28]. Bufadienolides from oocytes of *R. alata* were evaluated against kidney monkey Vero cells. Bufadienolides **1** (EC_50_ = 22.80 µM, S.I. > 5) and **7** (EC_50_ = 3.93 µM, S.I. = 2.7) present significant selectivity (Table 2). Although bufadienolide **7** shows a promising S.I., less cytotoxicity is required. On the contrary, the low cytotoxicity showed by bufadienolide **1** highlights its potential for further studies. Recently, 19-hydroxy-bufalin was evaluated against Vero cells and its low cytotoxicity, in comparison with bufadienolide **7**, suggests that hydroxyl at position 19 decreases cytotoxic effects against mammals [21]. Despite the potent anti-trypanosomal effect shown by compounds **3**, **5** and **6**, these bufadienolides were toxic to Vero cells, resulting in a reduced selectivity for the assayed parasite. In a previous study, hellebrigenin and telocinobufagin, two bufadienolides with anti-protozoal activity, were tested for cytotoxicity on mouse macrophages and erythrocytes, showing no toxic effects [19]. However, it has been found that rodent cells are over 1000-fold more resistant than human cells to the cytotoxic effects of cardiotonic sterols [29]. Differential cytotoxicity and water solubility are great attributes of potential anti-trypanosomals. In this context, microbial transformations carried out with bufadienolides **4**, **5** and **7** have produced nontoxic derivatives in human cells [30,31,32]. Nanoparticles containing bufadienolide **7** were injected in mice xenografted with human colon cancer cells, and their therapeutic potential was tested. The particles were able to inhibit the growth of tumor cells with efficient tumor targeting and cellular uptake [33].

Some bufadienolides have been found to be active against amphibian pathogens. Arenobufagin, γ-bufotalin and telocinobufagin isolated from the skin secretion of the boreal toad *Anaxyrus boreas* inhibit the harmful fungus *Batrachochytrium dendrobatidis* in vitro [34]. Amphibians are also affected by parasitic diseases caused by protozoa [35]. In fact, different families of anurans, including Bufonidae, are hosts of trypanosomes and are frequently parasitized by more than one species [36]. Blood samples from *R. alata* have not been examined for the presence of trypanosomes; however, congeneric species, as well as syntopic species such as the frog *Engystomops pustulosus*, are known to be parasitized by trypanosomes [37,38]. Trypanosomiasis causes anemia, food refusal, listlessness and hemorrhages with swollen lymph glands in amphibians. Some species of leeches (family Hirudinea) are considered the main aquatic vectors, while mosquitos, sand flies and midges are the terrestrial ones [37,38,39]. The occurrence of compounds **1–7** in the oocytes of toads is interesting. It has been determined that amphibians in the Bufonid family synthesize bufadienolides using cholesterol-marked isotopically [40]. These metabolites have been found in different organs of the animals, including parotoid glands, skin, plasma, bile, ovaries and oocytes [17,41,42,43]. Because they inhibit (Na^+^-K^+^)ATPase pump, bufadienolides very likely act as regulators of ionic equilibrium, and the high amounts of bufadienolides present in some species suggest they act as defenses against predators [44]. However, their role as antiparasitic metabolites should be taken into account.

## 3. Materials and Methods

### 3.1. General Experimental Procedures

One-dimensional and two-dimensional nuclear magnetic resonance (NMR) spectroscopy was performed on a Jeol instrument model Eclipse+ 400 (400 MHz) equipped with a 5 mm probe (Jeol, Peabody, MA, USA). NMR data was processed with the MestReNova software version 12.0.3-21384 (© Mestrelab Research S.L., Santiago de Compostela, A Coruña, Spain) and referenced based on the residual signal of deuterated solvents: chloroform (δ_H_ 7.26 and δ_C_ 77.00), dimethyl sulfoxide (δ_H_ 2.50 and δ_C_ 39.51) and methanol (δ_H_ 3.31 and δ_C_ 49.00). A digital polarimeter Jasco model P-2000 (Jasco, Easton, MD, USA) was used to determine optical rotations. The ultraviolet spectrophotometer Shimadzu model UV-2401 PC (Shimadzu, Columbia, MD, USA) was used to obtain the ultraviolet spectra. Infrared spectra were obtained using a Bruker Platinum ATR Alpha instrument (Bruker, Billerica, MA, USA). Melting points were measured using Stuart automatic melting point equipment, model SMP50 (Cole Parmer, Stone Staffordshire, UK).

High-performance liquid chromatography was performed on an Agilent 1100 HPLC with a Diode Array detector 1200 series (Agilent, Santa Clara, CA, USA). Mass spectra were acquired on a Bruker micrOTOF-QIII™ spectrometer (Bruker Daltonics, Billerica, MA) or in a Bruker MALDI-TOF-TOF Ultraflex (Bruker Daltonics, Billerica, MA, USA). For HR-ESI-Q-TOF analysis, MS spectra were obtained in positive ESI mode (50 to 2500 *m/z*), and the voltage of capillary set at 4500 V. Nitrogen was used as the nebulizer gas (2.0 bar, 200 °C and 9.0 L/min). The external calibration was performed with Agilent ESI-L Low Concentration Tuning Mix (Agilent Technologies, Santa Clara, CA, USA). Hexakis (1H, 1H, 2H-di-fluoro-ethoxy-phosphazene; *m/z* 622.0290 [M+H]^+^) (Synquest Laboratories, Alachua, FL, USA) was used as internal standard. For HR-MALDI-TOF analysis, the samples were transferred to an MTP plate (Bruker Daltonics, Billerica, MA, USA) and mixed with the matrix (α-cyano-4-hydroxy-cinammic acid, Sigma Aldrich, St. Louis, MO, USA). All experiments were performed in positive mode. Pepcalibstandard was used as an external calibrant (Bruker, Daltonics, Billerica, MA, USA).

### 3.2. Sample Collection

Ninety grams of oocytes were obtained from six gravid females of *R. alata* collected at Parque Nacional Soberanía, Republic of Panama (9.134994° N 79.722597° W). These gravid females spontaneously laid their oocytes when held in isolation inside plastic containers. The toads were collected under the authorization of the Ministry of Environment of Panama (Permit: SE/AQ-2-14) and released at the same collection site. The Ministry of Environment of Panama reviewed and approved the experimental protocols of the study before granting collection permits. All methods were carried out in accordance with relevant guidelines and regulations of the INDICASAT. All methods are reported in accordance with ARRIVE guidelines.

### 3.3. Extraction and Isolation of Bufadienolides

Freshly collected oocytes were macerated with 100 mL of chloroform-methanol (1:1) three times overnight in a shaker at room temperature. Extractions were pooled and dried under reduced pressure. The pool was suspended in 50 mL of deionized water and extracted with 50 mL of chloroform-methanol (2:1) six times. The organic phase was dried to provide 641.2 mg of residue. This extract was loaded onto Supelclean^TM^ LC-18 SPE cartridges (Supelco, Bellefonte, PA, USA) and fractionated by employing a stepwise gradient of methanol in water at 40, 80 and finally 100% of methanol to produce fractions 1–3, respectively. The solvent was removed by rotary evaporation under a vacuum, and the remaining water was removed by lyophilization. Fraction 3 was diluted in methanol, and the compounds were separated by HPLC. We used a semipreparative Synergy Hydro C18 column (250 × 10 mm, 4 µm, 80 Å) (Phenomenex, Torrance, CA, USA). The mobile phase consisted of water with 0.1% trifluoroacetic acid (TFA) (Solvent A) and a mixture of methanol–acetonitrile (1:1) acidified with TFA at 0.1% (Solvent B). Elution detection was performed at λ 300 nm at a flow rate of 1 mL/min. The fractions were collected individually and as result the isolation of compounds **1** (5.7 mg, Rt 42.1 min), **2** (8.3 mg, Rt 63.0 min), **3** (1.7 mg, Rt 65.8 min), **4** (3.4 mg, Rt 67.5 min), **5** (4.4 mg, Rt 70.7 min), **6** (2.1 mg, Rt 75.4 min) and **7** (2.4 mg, Rt 77.1 min) was achieved. The purity of all compounds was found to be higher than 95%, as analyzed by ^1^H NMR spectroscopy.

### 3.4. Compounds Characterization Data

16β-hydroxyl-hellebrigenin (**1**): yellow solid; mp 81 °C; for ^1^H and ^13^C NMR, data see Supplementary Information File; HR-ESI-MS *m/z* 433.2222 [M + H]^+^ (calculated for C_24_H_33_O_7_, 433.2221).

Desacetyl-bufotalin (**2**): yellow solid; mp 71 °C; for ^1^H and ^13^C-NMR data, see Supplementary Information File. HR-ESI-MS *m/z* 403.2472 [M + H]^+^ (calculated for C_24_H_35_O_5_, 403.2479).

19-formyl-dyscinobufotalin (**3**): white solid; [α]${}_{D}^{16}$= + 11° (0.001 *c*, methanol); UV (CH_3_OH) λ_max_ (log ɛ) 201 nm (4.45) 300 nm (3.90); IR υ_max_ 3589, 2864 and 1715 cm^−1^; for ^1^H and ^13^C NMR data, see Table 1 and spectra in Supplementary Information (Figures S2–S7); HR-MALDI-MS *m/z* 487.2283 [M + H]^+^ (calculated for C_27_H_35_O_8_, 487.2326) (Figure S8).

Bufotalin (**4**): white solid; mp 79 °C; For ^1^H and ^13^C-NMR data, see Supplementary Information File. HR-ESI-MS *m/z* 445.2616 [M + H]^+^ (calculated for C_26_H_37_O_6_, 445.2585).

Cinobufotalin (**5**): yellow solid; mp 81 °C; For ^1^H and ^13^C-NMR data, see Supplementary Information File. HR-ESI-MS *m/z* 459.2385 [M + H]^+^ (calculated for C_26_H_35_O_7_, 459.2377).

Dyscinobufotalin (**6**): white solid; for ^1^H and ^13^C-NMR data, see Supplementary Information File. HR-ESI-MS *m/z* 473.2518 [M + H]^+^ (calculated for C_27_H_37_O_7_, 473.2534).

Bufalin (**7**): white solid; For ^1^H and ^13^C-NMR data, see Supplementary Information File. HR-ESI-MS *m/z* 387.2512 [M + H]^+^ (calculated for C_24_H_35_O_4_, 387.2530).

### 3.5. Anti-Trypanosomal Activity

Anti-trypanosomal bioassays were performed using the recombinant strain Tulahuen, clone C4, of *T. cruzi* in the form of trypomastigotes (ATCC, Manassas, VA, USA). This strain expresses the enzyme β-galactosidase [45]. The parasites were incubated at 37 °C under an atmosphere of 5% CO_2_ in RPMI-1640 culture medium supplemented with L-glutamine,4-(2-hydroxyethyl)-piperazine-1-ethanesulfonic acid (HEPES) buffer, NaHCO_3_, 10% FBS and 0.05% gentamicin (50 mg/mL). Epithelial kidney monkey Vero cells (ATCC, Manassas, VA, USA) were incubated for 24 h and then infected with the parasites 24 h prior to the addition of the compounds. After additional 24 h of infection, the compounds (**1–7**) were dissolved in DMSO and tested at 10, 2, 0.4 and 0.08 µg/mL for five days. The drug benznidazole was used as a positive control. Chlorophenol-red-β-D-galactopyranoside (Roche Applied Science) was added to each well and then allowed to react with the β-galactosidase of the rest of the living parasites for 4.5 h to quantify the biological activity. Absorbance was measured at 570 nm using a plate reader (Sinergy HT, BioTek Instruments Inc., Winooski, VT, USA).

### 3.6. Cytotoxicity Assay

Epithelial kidney monkey Vero cells were incubated at 37 °C in 96-well plates under an atmosphere of 5% CO_2_, using RPMI-1640 medium (Sigma-Aldrich, St. Louis, MO, USA) supplemented with 0.05% gentamicin (50 mg/mL) and 10% FBS (fetal bovine serum; Gibco, Invitrogen, Carlsbad, CA, USA). Cells were allowed to adhere for one day before incubation for five days with the compounds. Doxorubicin and DMSO were used as positive and negative controls, respectively. When the incubation period ended, 3-(4, 5-di-methyl-thiazol-2-yl)-2, 5-di-phenyl-tetra-zolium bromide (MTT) was added to the wells, and after 4 h, absorbance was measured at 570 nm using a color plate reader. Cytotoxicity was assessed colorimetrically by calculating the capacity of the remaining Vero cells to reduce the yellow MTT into the dark purple formazan product, as reported [46].

### 3.7. Statistical Analysis of Bioassays

All bioassays represent independent analysis and were carried out in duplicate. The data analysis complement Wizard of Excel 2000 (Microsoft, Seattle, WA, USA) was used for the statistical analysis of the 50% inhibiting (IC_50_) and cytotoxic (CC_50_) concentration values by adjusting the dose-response curve to a sigmoidal model.

### 3.8. Molecular Docking

Molecular docking analysis was performed with the Molecular Operating Environment (MOE), software version 2018.01 (Chemical Computing Group, Montreal, QC, Canada). Molecular docking was carried out by allowing ligands to move, keeping proteins rigid. Cruzipains and ligands were prepared with the standard protocol of MOE. The X-ray crystallographic data of twenty-four proteins were extracted from the Protein Data Bank. The structures of the proteins were corrected on their misallocated amino acid residues, protonated in 3D, and the energies were minimized using the MMFF94x force field. Downloaded proteins were aligned before the docking simulation. All cruzipains showed protein similarity from 99.1 to 100%.

Validation of the protocol was carried out by redocking the cruzipain 1EWO [47], 2AIM [48], 1U9Q [49] and 4XUI [50] (resolution between 2.10–2.51 Å); with their respective co-crystallized ligands VSC, ZRA, 186 and 2VC. Co-crystallized ligands were subjected to a simulated auto-mode coupling-induced fit. The binding pocket was defined as the set of amino acid residues within 4.5 Å from the co-crystallized ligand. Bufadienolides (**1**, **3, 7**) were selected for molecular docking with cruzipain since they present the highest bioactivity and significant selectivity in vitro. The docking conformations that showed the lowest docking scores were selected for analysis.

## 4. Conclusions

Seven steroids from the bufadienolide class were isolated from the oocytes of the toad *R. alata*. Among them, 19-formyl-dyscinobufotalin (**3**) is reported as a new natural product. While compound **2** was the only one reported as inactive, new bufadienolide **3** showed the most potent anti-trypanosomal activity, even more active than the control drug benznidazole. The in vitro assays and structural–activity relationship analysis suggest the formyl group at C-19 and esterification of the hydroxy group at C-16 increase anti-trypanosomal activity. 16β-hydroxyl-hellebrigenin showed significant selectivity and a promising therapeutic window. Furthermore, molecular docking analysis revealed bufadienolides **1**, **3** and **7** interact with the main protease from *T. cruzi*, cruzipain, at the amino acid residues GLN-19, ASP-158, HIS-159 and TRP-177 through H-bonds. Chemical interactions involve the group’s hydroxyl at positions C-5 and C-14 and formyl at C-19 at the cruzipain active site. Given the lack of therapeutic options to currently treat American trypanosomiasis, this work can serve as the basis for further studies that aim for the development of bufadienolides or their derivatives as drugs against Chagas disease.

## Figures and Tables

**Table 1:** NMR (^1^H 400 MHz, ^13^C 100 MHz) data for compound **3** in methanol-d_4_ (δ in ppm, *J*, in Hz).

No.	^13^C, Type	^1^H, Mult. (*J* in Hz)
**1a**	18.6, CH_2_	2.26, m
**1b**		1.77, m
**2a**	27.5, CH_2_	1.77, m
**2b**		1.77, m
**3**	68.0, CH	4.15, m
**4a**	36.7, CH_2_	1.99, m
**4b**		1.61, m
**5**	75.8, C	
**6a**	38.3, CH_2_	2.23, m
**6b**		1.49, m
**7a**	24.1, CH_2_	1.77, m
**7b**		1.11, m
**8**	43.1, CH	1.77, m
**9**	34.4, CH	2.47, td (3.8, 12.2)
**10**	56.0, C	
**11a**	22.9, CH_2_	1.66, m
**11b**		1.39, m
**12a**	40.5, CH_2_	1.77, m
**12b**		1.49, m
**13**	46.0, C	
**14**	73.1, C	
**15**	60.7, CH	3.75, m
**16**	76.3, CH	5.47, m
**17**	51.2, CH	2.93, d (9.2)
**18**	17.3, CH_3_	0.81, s
**19**	210.1, CH	10.00 s
**20**	118.2, C	
**21**	153.6, CH	7.36, m
**22**	150.9, CH	8.02, m
**23**	114.2, CH	6.24, dd (0.8, 9.7)
**24**	164.0, C=O	
**1’**	174.9, C=O	
**2’a**	28.1, CH_2_	2.23, m
**2’b**		2.11, m
**3’**	9.3, CH_3_	0.94, t (7.8)

**Table 2:** Anti-trypanosomal activity and mammalian cytotoxic of bufadienolides **1**-**7** isolated from the oocytes of *Rhinella alata*.

Compound	*T. cru* *zi*	VERO CELL	Selectivity (CC_50_/EC_50_)
	EC_50_ (µM)	CC_50_ (µM)	
**1**	22.80 ± 4.11	>135	>5
**2**	Inactive	>28	----
**3**	0.19 ± 0.07	0.22 ± 0.09	1.16
**4**	19.60 ± 2	>46	>2
**5**	27.80 ± 3.77	1.88 ± 0.20	0.07
**6**	12.30 ± 1.87	0.02 ± 0.002	<0.01
**7**	3.93 ± 0.57	10.61 ± 1.54	2.69
Benznidazole	2.73 ± 0.10	51.36 ± 6.0	18.81
Doxorubicin	Not tested	0.23	----

**Table 3:** Calculations for molecular docking of bufadienolides with cruzipain.

Bufadienolide	RMSD (Å)	Docking Score	Ligand Group	Amino Acid Residue	Energy (kcal/mol)	Distance (Å)
1	2.5812	−4.2777	CH=O at 19	GLN-19	−1.5	2.32
			OH at 14	ASP-158	−1.7	2.10
3	2.0099	−5.3984	CH=O at 19	GLN-19	−2.2	2.09
			CH=O at 19	TRP-177	−0.5	2.59
			OH at 5	HIS-159	−1.5	2.35
7	3.0381	−4.1206	OH at 14	ASP-158	+1.3	1.95

## Data Availability

All data are available in the main text or the Supplementary Materials.

## Supplementary Materials

The following supporting information can be downloaded at: https://www.mdpi.com/article/10.3390/molecules29010196/s1, Figure S1: RP-HPLC chromatogram profile of fraction-3 from oocytes of *Rhinella alata*; Figure S2: ^1^H-NMR spectrum of 19-formyl-dyscinobufotalin (**3**); Figure S3: ^13^C-NMR spectrum of 19-formyl-dyscinobufotalin (**3**); Figure S4: DEPT spectrum comparison of 19-formyl-dyscinobufotalin (**3**); Figure S5: HMBC spectrum of 19-formyl-dyscinobufotalin (**3**); Figure S6: HSQC spectrum of 19-formyl-dyscinobufotalin (**3**); Figure S7: COSY spectrum of 19-formyl-dyscinobufotalin (**3**); Figure S8: HR-MALDI-MS spectrum of 19-formyl-dyscinobufotalin (**3**); Figure S9: Alignment and superposition of 24 cruzipains and co-crystallized ligands; Table S1: The binding affinity values of compounds **1–7**; NMR data for compounds **1**, **2**, **4–7** isolated from the oocytes of *Rhinella alata*; Figure S10: ^1^H NMR spectrum for compound 16β-hydroxyl-hellebrigenin (**1**); Figure S11: ^1^H NMR spectrum for compound desacetyl-bufotalin (**2**); Figure S12: ^1^H NMR spectrum for compound bufotalin (**4**); Figure S13: ^1^H NMR spectrum for compound cinobufotalin (**5**); Figure S14: ^1^H NMR spectrum for compound dyscinobufotalin (**6**); Figure S15: ^1^H NMR spectrum for compound bufalin (**7**); Supplementary references.
