# Supplementary material for: Anti-Trypanosomal Bufadienolides from the Oocytes of the Toad Rhinella alata (Anura, Bufonidae)
Source: Molecules. 2023 Dec 29;29(1):196. doi: 10.3390/molecules29010196 (PMC10779871; doi:10.3390/molecules29010196)
Supplement: Supplementary file 1 [file molecules-29-00196-s001.zip › molecules-2782939-supplementary.pdf]

Antitrypanosomal bufadienolides from the oocytes of the toad *Rhinella alata* (Anura, Bufonidae)

*Candelario Rodriguez, Roberto Ibáñez, Dionisio A. Olmedo, Michelle NG, Carmenza Spadafora, Armando A. Durant-Archibold and Marcelino Gutiérrez**

Supporting information

Contents **Page**

Figure S1. RP-HPLC chromatogram profile of fraction-3 from oocytes of *Rhinella alata* 2
Figure S2. ^{1}H NMR spectrum of 19-formyl-dyscinobufotalin (**3**) 3
Figure S3. ^{13}C NMR spectrum of 19-formyl-dyscinobufotalin (**3**) 4
Figure S4. DEPT spectrum comparison of 19-formyl-dyscinobufotalin (**3**) 5
Figure S5. HMBC spectrum of 19-formyl-dyscinobufotalin (**3**) 6
Figure S6. HSQC spectrum of 19-formyl-dyscinobufotalin (**3**) 7
Figure S7. COSY spectrum of 19-formyl-dyscinobufotalin (**3**) 8
Figure S8. HR-MALDI-MS spectrum of 19-formyl-dyscinobufotalin (**3**) 9
Figure S9. Alignment and superposition of 24 cruzipains and cocrystallized ligands 10
Table S1. The binding affinity values of compounds **1-7** 11
NMR data for compounds **1, 2, 4-7** isolated from the oocytes of *Rhinella alata* 12
Figure S10. ^{1}H NMR spectrum for compound 16β-hydroxyl-hellebrigenin (**1**) 14
Figure S11. ^{1}H NMR spectrum for compound desacetyl-bufotalin (**2**) 15
Figure S12. ^{1}H NMR spectrum for compound bufotalin (**4**) 16
Figure S13. ^{1}H NMR spectrum for compound cinobufotalin (**5**) 17
Figure S14. ^{1}H NMR spectrum for compound dyscinobufotalin (**6**) 18
Figure S15. ^{1}H NMR spectrum for compound bufalin (**7**) 19
References 20

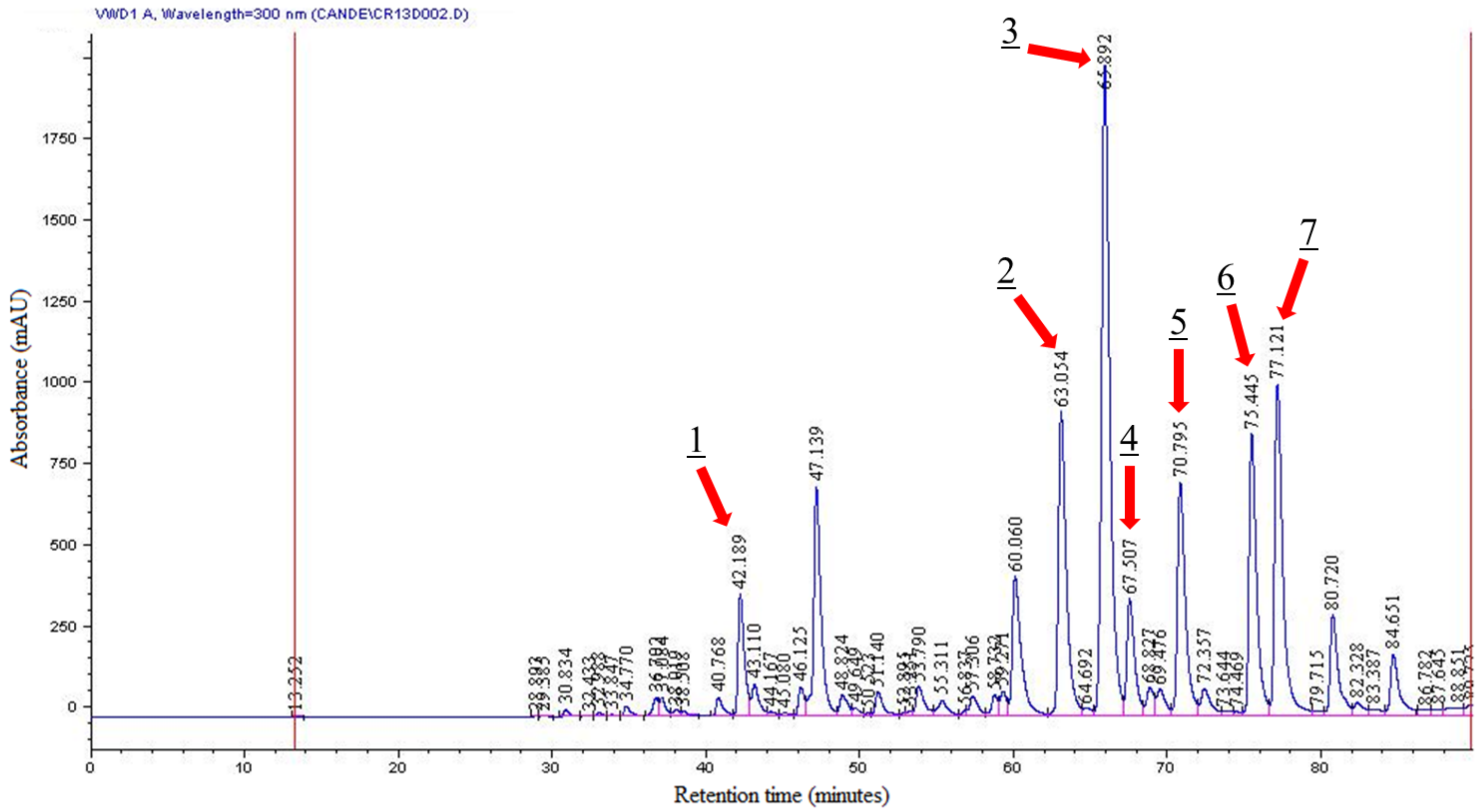

Figure S1. RP-HPLC chromatogram profile of fraction-3 from oocytes of *Rhinella alata*

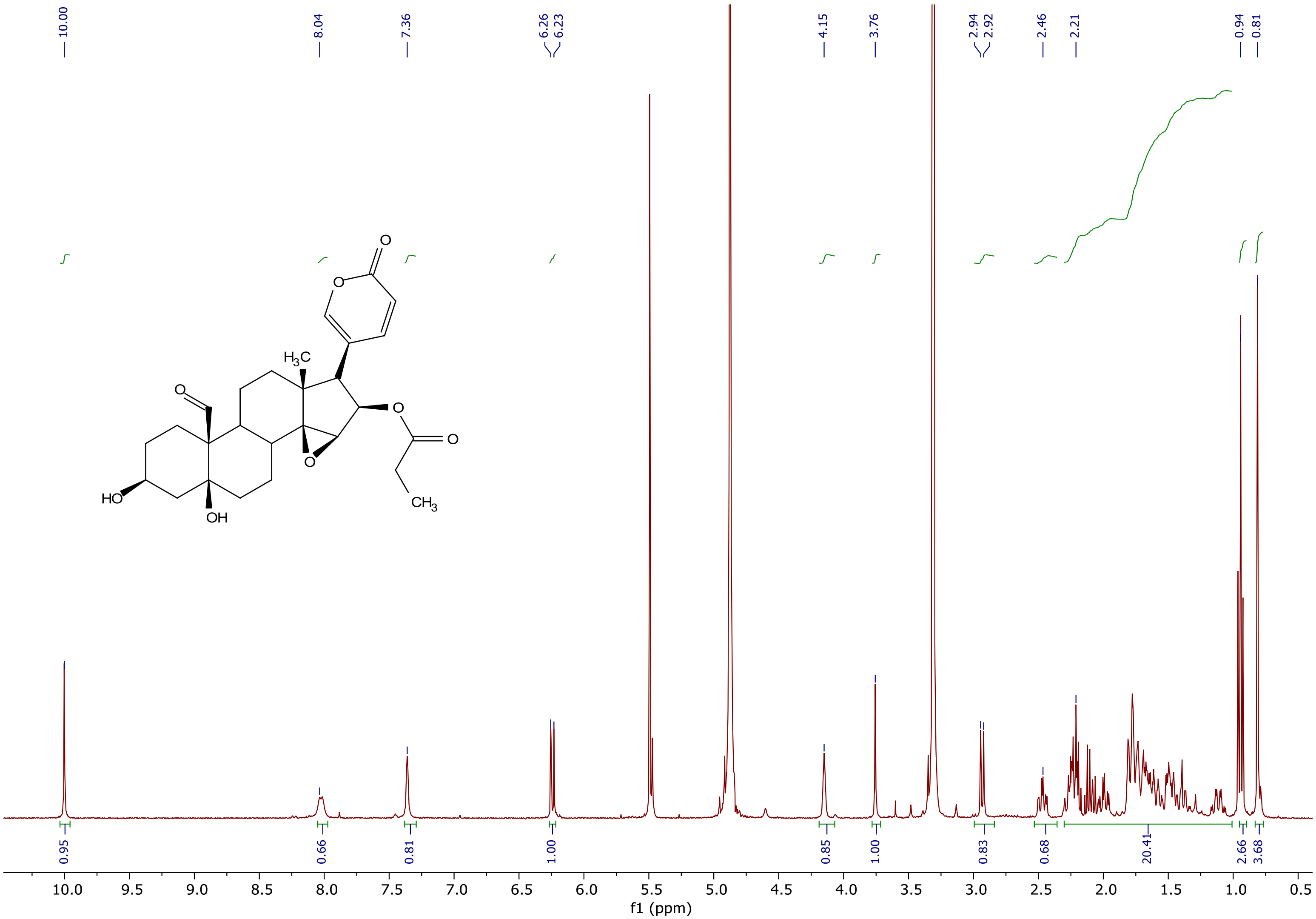

Figure S2. ^{1}H NMR spectrum (400 MHz in methanol-d_4) of 19-formyl-dyscinobufotalin (**3**)

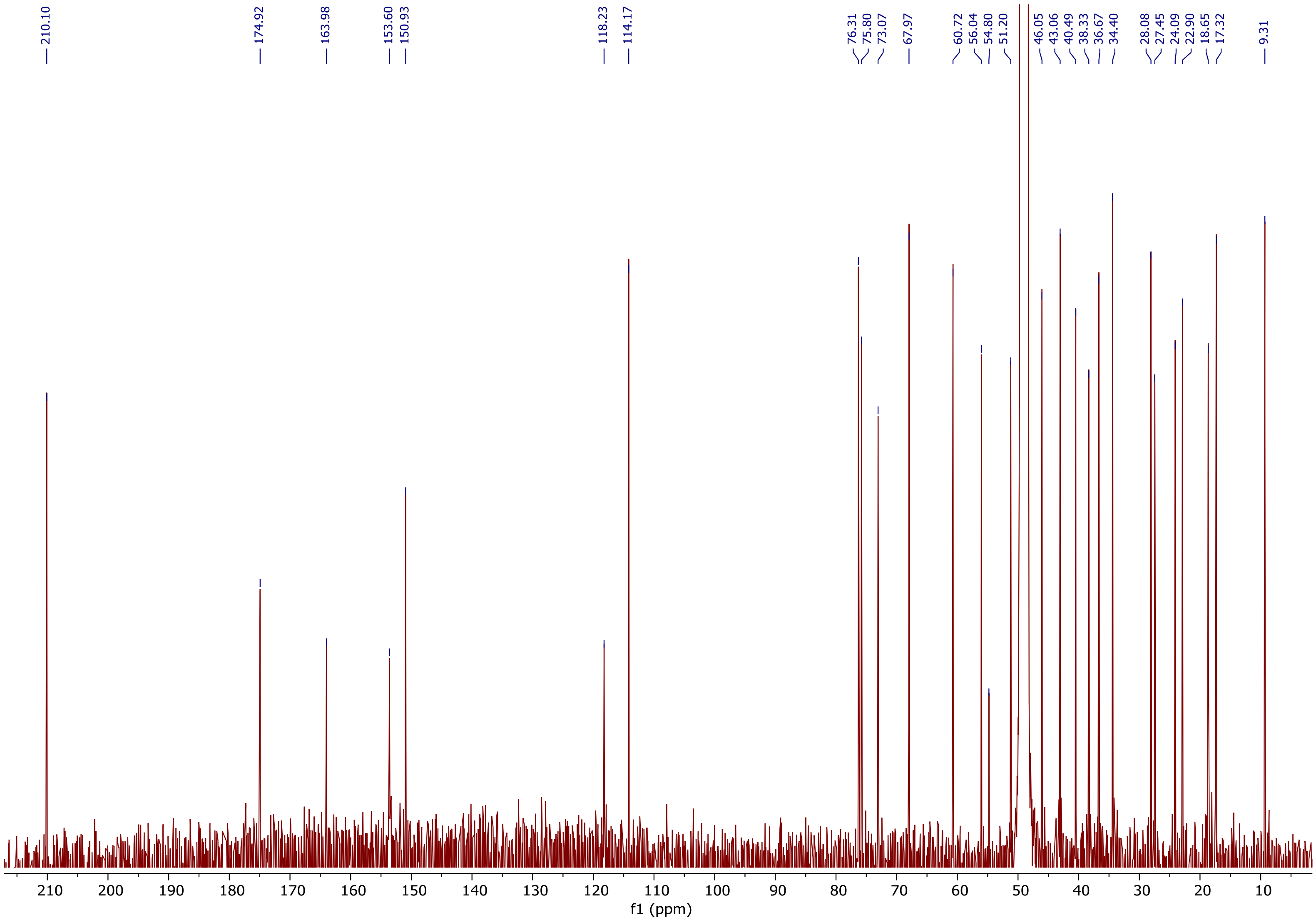

Figure S3. ^{13}C NMR spectrum (100 MHz in methanol-d_4) of 19-formyl-dyscinobufotalin (**3**)

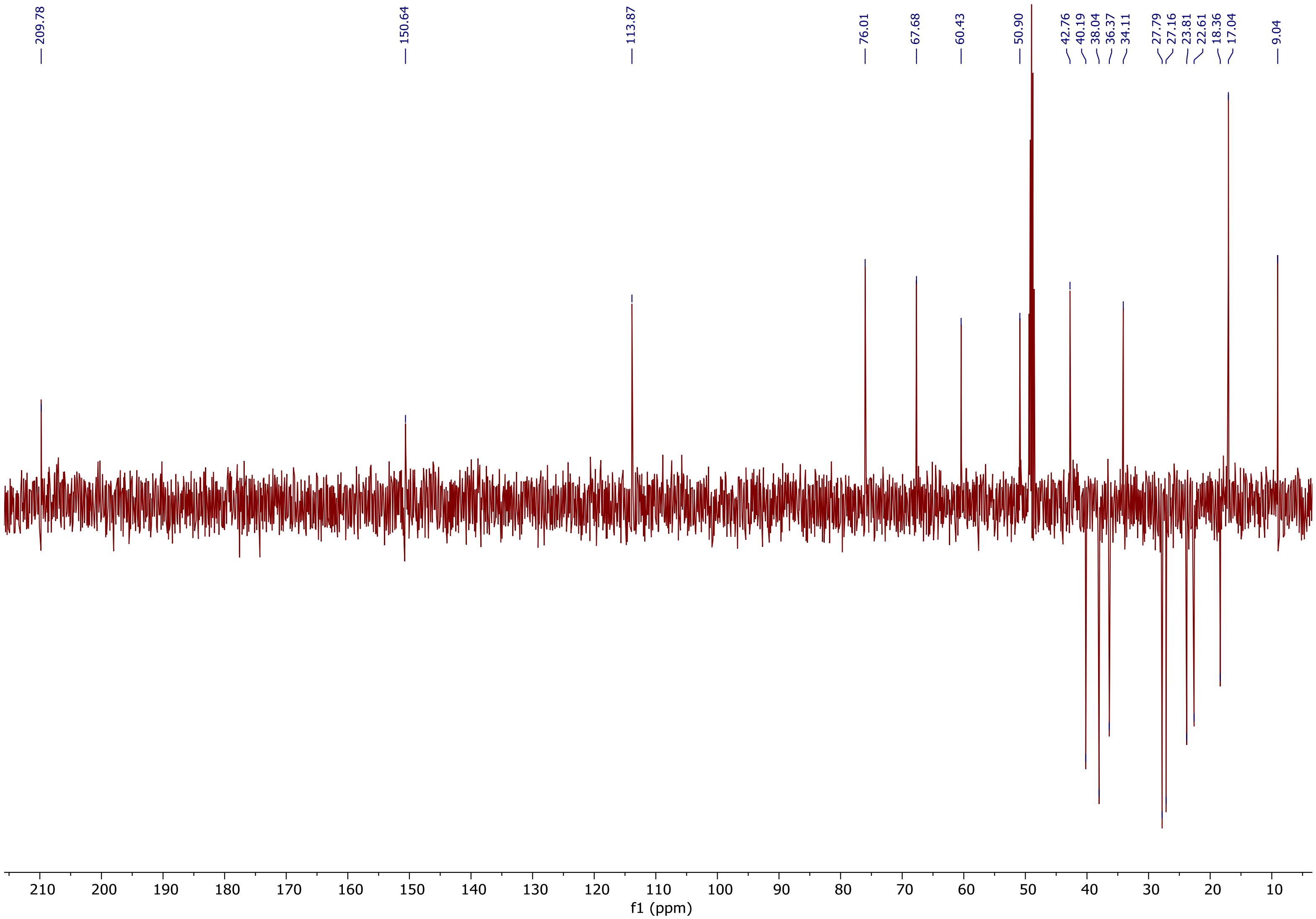

Figure S4. DEPT-135 NMR spectrum (in methanol-d$_4$) of 19-formyl-dyscinobufotalin (**3**)

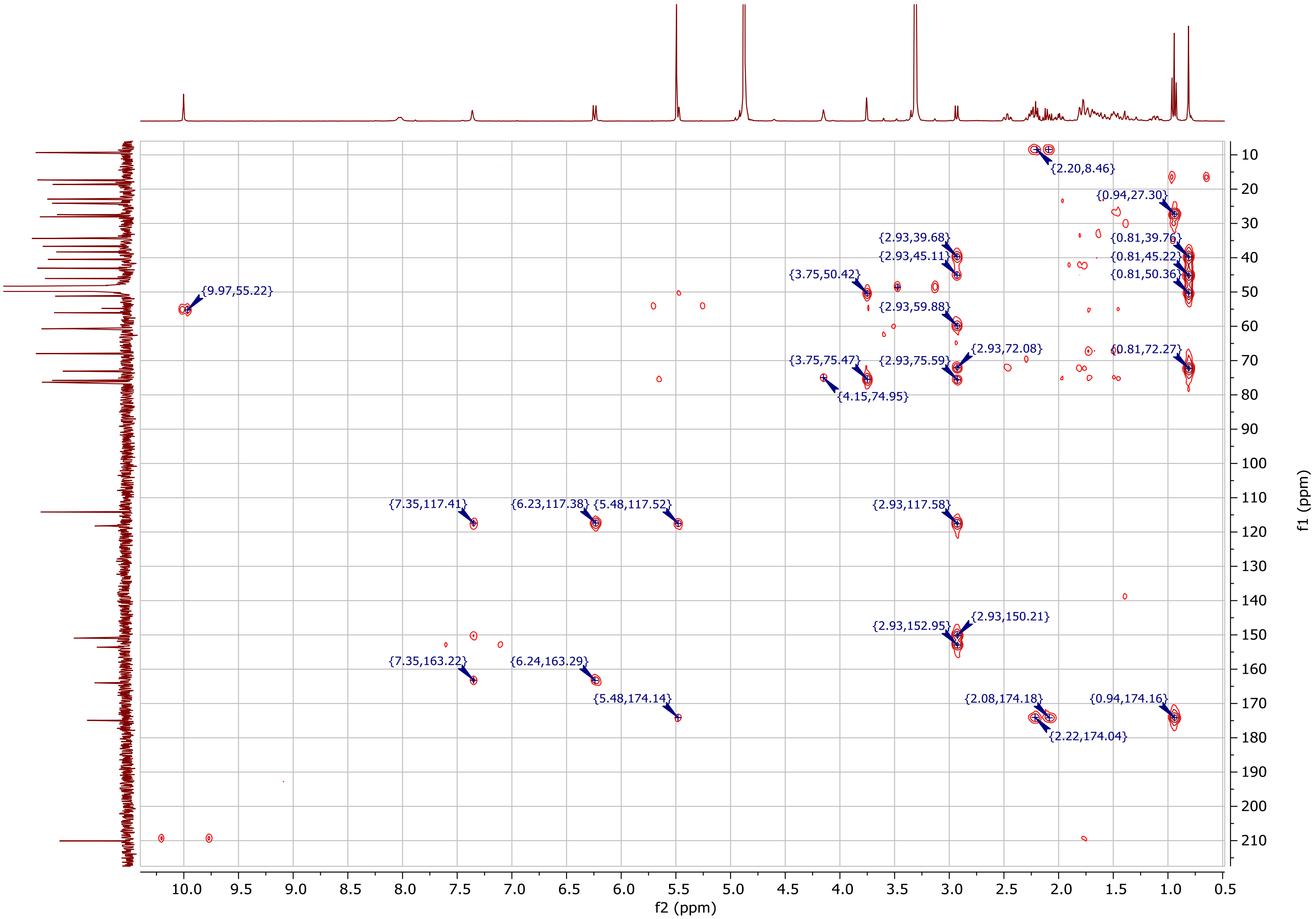

Figure S5. HMBC NMR spectrum (in methanol-d$_4$) of 19-formyl-dyscinobufotalin (**3**)

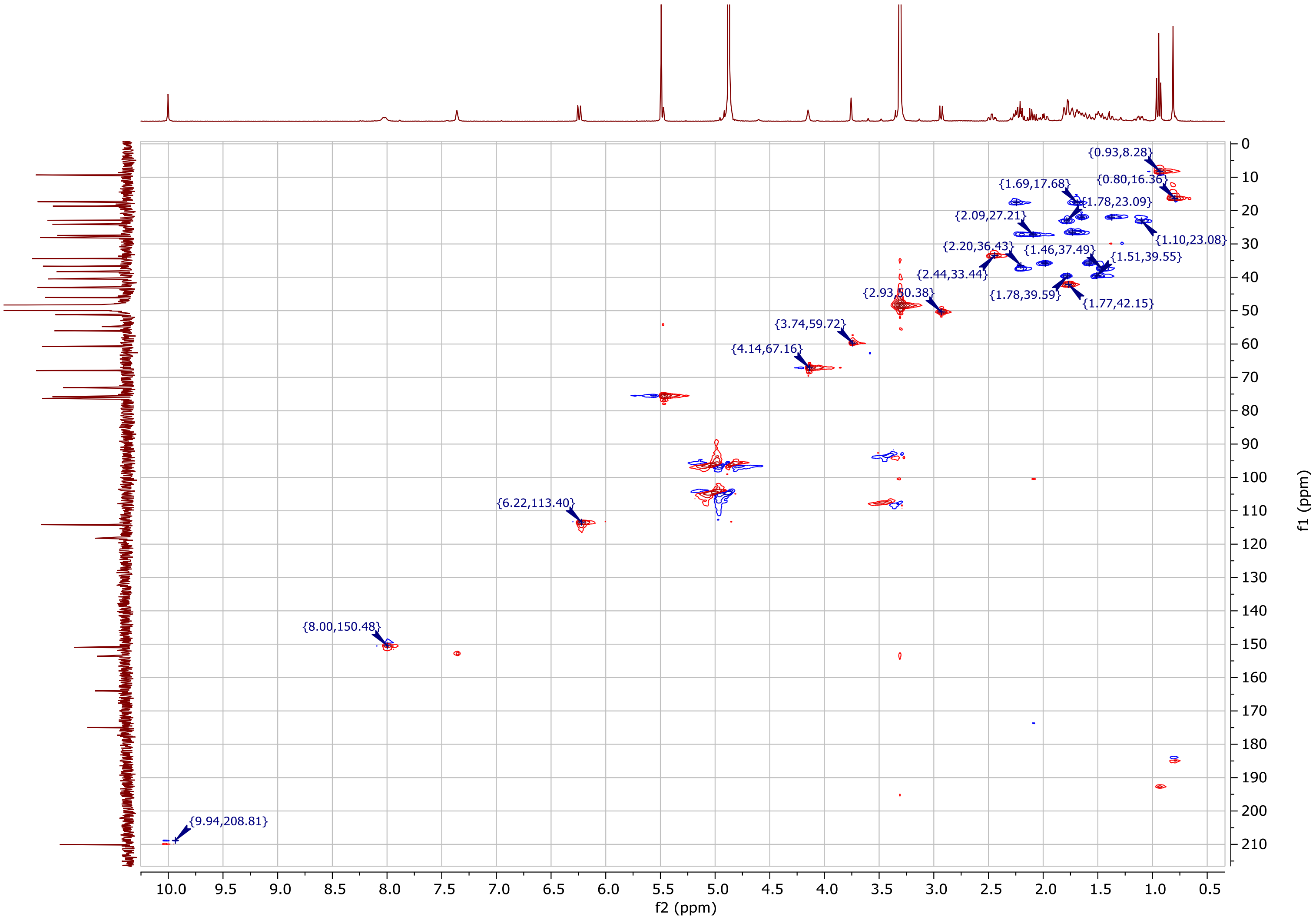

Figure S6. HSQC NMR spectrum (in methanol-d_4) of 19-formyl-dyscinobufotalin (**3**)

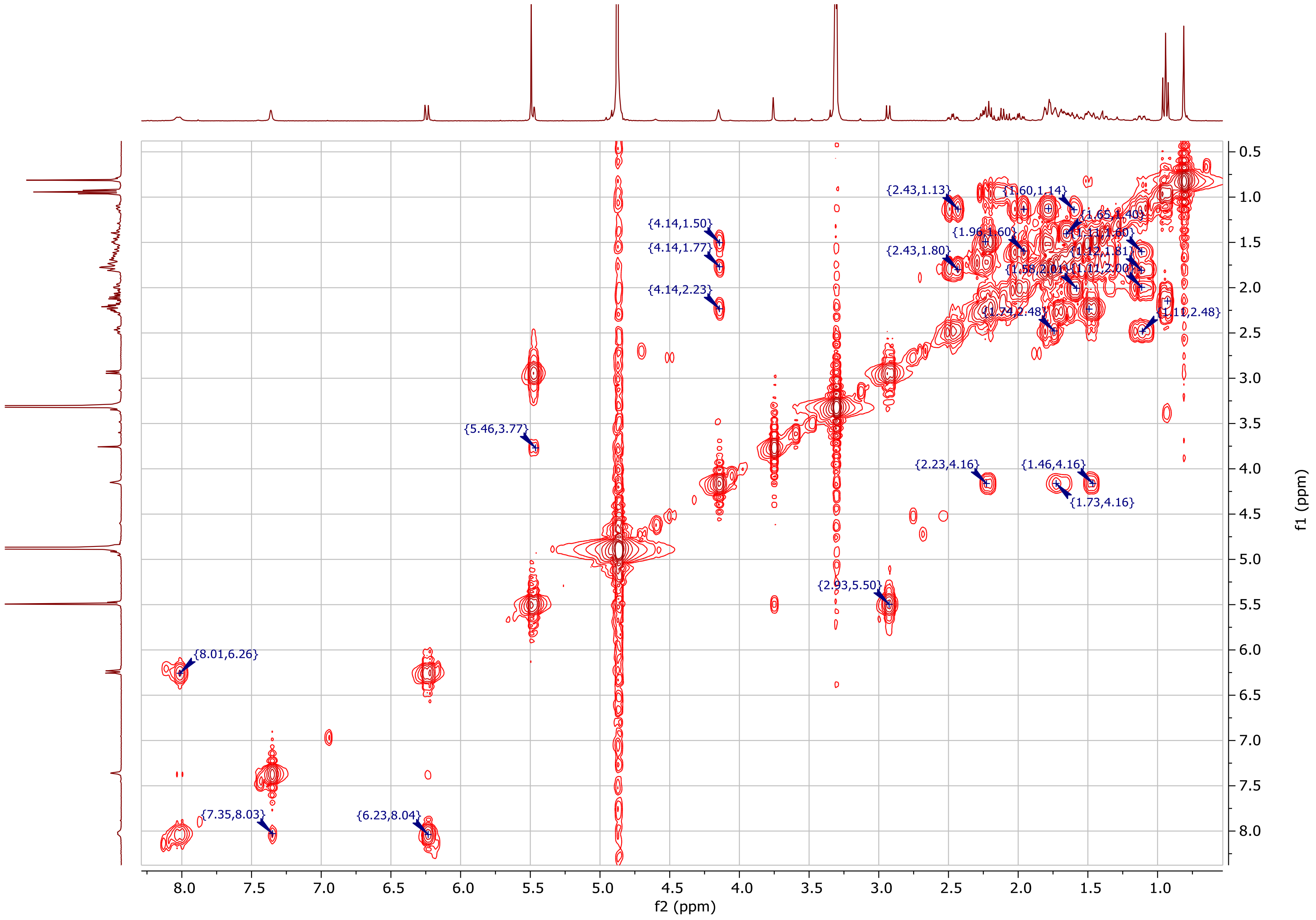

Figure S7. COSY NMR spectrum (in methanol-d_4) of 19-formyl-dyscinobufotalin (**3**)

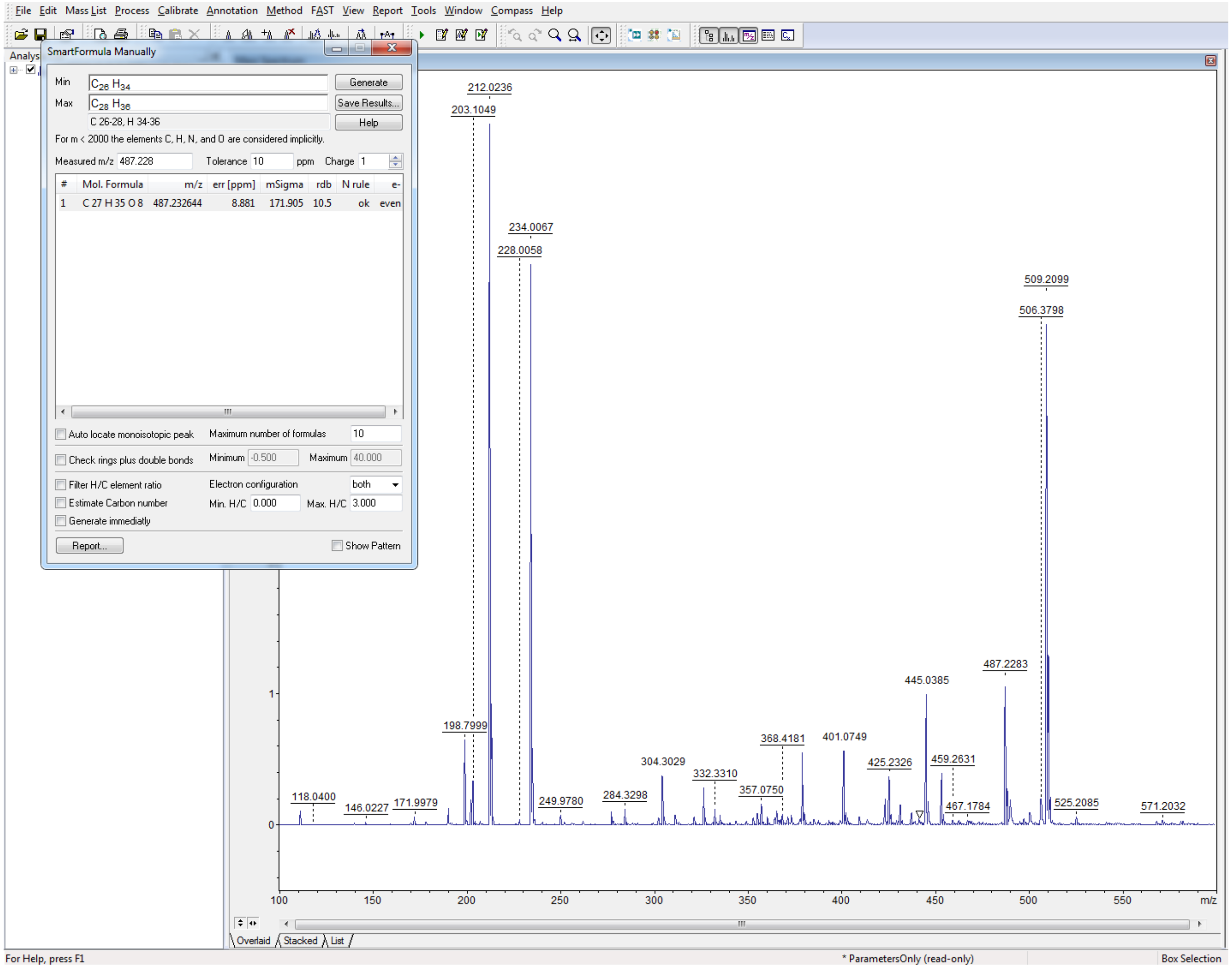

Figure S8. HR-MALDI-MS spectrum of 19-formyl-dyscinobufotalin (**3**)

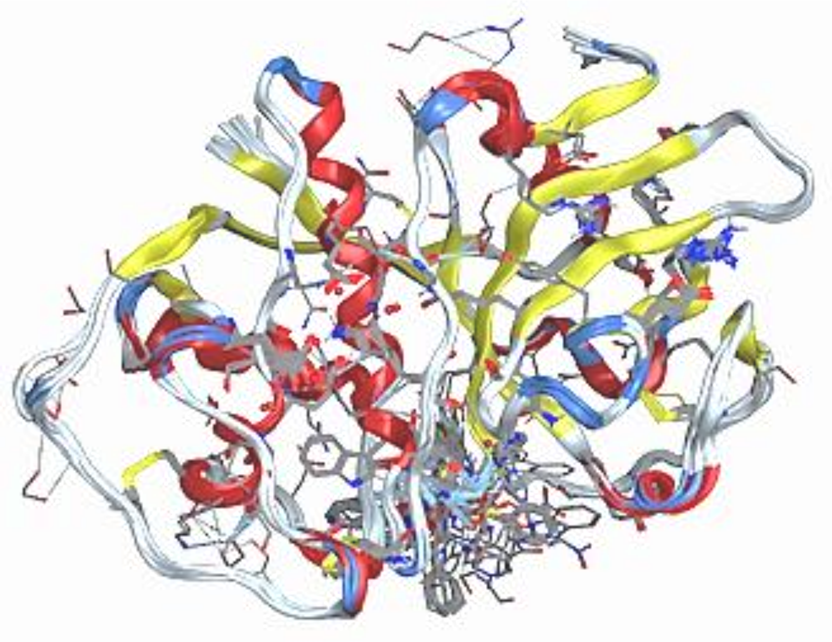

Figure S9. Alignment and superposition of 24 cruzipains and co-crystallized ligands obtained from the Protein Data Bank.

Table S1. Binding affinity values of compounds **1-7**

Compound	Binding Energy (Kcal/mol)	
	PDB ID: 1EWO	*PDB ID: 2AIM*
**1**	-1.5	-3.0
**2**	-4.7	-5.4
**3**	-6.1	-2.2
**4**	-1.1	-1.1
**5**	-2.7	-1.5
**6**	-2.9	-1.5
**7**	-2.0	-1.2

NMR data for bufadienolides **1, 2, 4-7** isolated from the oocytes of *Rhinella alata*

16β-hydroxyl-hellebrigenin (**1**) [1]: ^{1}H-NMR (methanol-d4, 400 MHz) δ: 8.12 (1H, dd, *J* = 2.4, 9.7, H-22), 7.44 (1H, dd, *J* = 0.8, 2.4 H-21), 6.19 (1H, dd, *J* = 0.8, 9.8, H-23), 4.50 (1H, t, *J* = 6.8, H-16), 4.15 (1H, m, H-3), 2.75 (1H, d, *J* = 8.3, H-17), 2.51 (1H, dd, *J* = 7.3, 14.6, H-15a), 1.76 (1H, m, H-15b), 10.05 (1H, s, H-19), 0.75 (3H, s, H-18). ^{13}C-NMR (Methanol-d4, 100 MHz) δ: 210.2 (CH, C-19), 165.1 (C=O, C-24), 152.9 (CH, C-22), 151.9 (CH, C-21), 120.4 (C, C-20), 113.0 (CH, C-23), 85.6 (C, C-14), 75.9 (C, C-5), 73.4 (CH, C-16), 68.1 (CH, C-3), 59.4 (CH, C-17), 56.3 (C, C-10), 50.2 (C, C-13), 43.0 (CH, C-8), 42.4 (CH_2, C-15), 41.8 (CH_2, C-12), 40.5 (CH, C-9), 38.7 (CH_2, C-4), 37.5 (CH_2, C-6), 27.7 (CH_2, C-2), 25.3 (CH_2, C-7), 23.4 (CH_2, C-11), 18.6 (CH_2, C-1), 17.2 (CH_3, C-18).

Desacetyl-bufotalin (**2**) [2]: yellow solid; ^{1}H-NMR (DMSO-d, 400 MHz) δ: 8.08 (1H, dd, *J* = 2.4, 9.8, H-22), 7.47 (1H, d, *J* = 1.9, H-21), 6.12 (1H, d, *J* = 9.8, H-23), 4.42 (1H, t, *J* = 7.8, H-16), 3.89 (1H, br s, H-3), 2.63 (1H, d, *J* = 8.2, H-17), 2.44 (1H, dd, *J* = 8.3, 14.6, H-15a), 1.60 (1H, d, *J* = 14.1, H-15b), 0.84 (3H, s, H-19), 0.64 (3H, s, H-18). ^{13}C-NMR (DMSO-d, 100 MHz) δ: 161.5 (C=O, C-24), 151.3 (CH, C-22), 150.1 (CH, C-21), 118.6 (C, C-20), 110.9 (CH, C-23), 83.1 (C, C-14), 70.5 (CH, C-16), 64.6 (CH, C-3), 57.5 (CH, C-17), 48.7 (C, C-13), 42.6 (CH_2, C-15), 41.3 (CH, C-8), 39.7 (CH_2, C-12), 35.6 (CH, C-5), 34.8 (C, C-10), 34.6 (CH, C-9), 33.0 (CH_2, C-4), 29.5 (CH_2, C-1), 27.5 (CH_2, C-11), 26.4 (CH_2, C-6), 23.7 (CH_3, C-19), 20.9 (CH_2, C-2), 20.8 (CH_2, C-7), 16.8 (CH_3, C-18).

Bufotalin (**4**) [2]: white solid; ^{1}H-NMR ($CDCl_3$, 400 MHz) δ: 8.02 (1H, dd, *J* = 2.9, 9.7, H-22), 7.21 (1H, m, H-21), 6.18 (1H, d, *J* = 9.7, H-23), 5.51 (1H, m, H-16), 4.14 (1H, m, H-3), 2.85 (1H, d, *J* = 8.7, H-17), 0.93 (3H, s, H-19), 0.76 (3H, s, H-18). ^{13}C-NMR ($CDCl_3$, 100 MHz) δ: 170.3 (C=O, C-1’), 162.3 (C=O, C-24), 151.1 (CH, C-21), 149.4 (CH, C-22), 117.1 (C, C-20), 113.3 (CH, C-23), 84.6 (C, C-14), 73.7 (CH, C-16), 67.1 (CH, C-3), 57.3 (CH, C-17), 49.6 (C, C-13), 42.5 (CH, C-8), 41.0 (CH_2, C-12), 40.6 (CH_2, C-15), 36.0 (CH, C-5), 35.7 (CH, C-9), 35.5 (C, C-10), 33.4 (CH_2, C-4), 29.7 (CH_2, C-1), 28.0 (CH_2, C-2), 26.5 (CH_2, C-6), 23.9 (CH_3, C-19), 21.3 (CH_2, C-7), 21.2 (CH_2, C-11), 21.2 (CH_3, C-2’), 16.6 (CH_3, C-18).

Cinobufotalin (**5**) [3]: yellow solid; ^{1}H-NMR ($CDCl_3$, 400 MHz) δ: 7.91 (1H, m, H-22), 7.14 (1H, m, H-21), 6.20 (1H, d, *J* = 9.9, H-23), 5.42 (1H, d, *J* = 9.1, H-16), 4.21 (1H, br s, H-3), 2.78 (1H, d, *J* = 9.6, H-17), 1.87 (3H, s, H-2’), 0.96 (3H, s, H-19), 0.79 (3H, s, H-18). ^{13}C-NMR ($CDCl_3$, 100 MHz) δ: 170.4 (C=O, C-1’), 162.0 (C=O, C-24), 151.5 (CH, C-21), 148.5 (CH, C-22), 116.3 (C, C-20), 114.1 (CH, C-23), 75.1 (C, C-5), 74.7 (CH, C-16), 72.5 (C, C-14), 68.3 (CH, C-3), 59.6 (CH, C-15), 50.3 (CH, C-17), 45.2 (C, C-13), 42.8 (CH, C-9), 41.0 (C, C-10), 40.2 (CH_2, C-12), 36.9 (CH_2, C-4), 34.5 (CH_2, C-6), 32.3 (CH, C-8), 28.0 (CH_2, C-2), 24.9 (CH_2, C-1), 23.2 (CH_2, C-7), 21.5 (CH_2, C-11), 20.7 (CH_3, C-2’), 17.3 (CH_3, C-18), 16.9 (CH_3, C-19).

Dyscinobufotalin (**6**) [4]: white solid; ^{1}H-NMR ($CDCl_3$, 400 MHz) δ: 7.91 (1H, m, H-22), 7.14 (1H, m, H-21), 6.20 (1H, d, *J* = 10, H-23), 5.43 (1H, dd, *J* = 0.9, 9.2, H-16), 4.22 (1H, m, H-3), 2.79 (1H, d, *J* = 9.2, H-17), 0.96 (CH_3, s, H-19), 0.95 (3H, t, *J* = 7.5, H-3'), 0.80 (CH_3, s, H-18). ^{13}C-NMR ($CDCl_3$, 100 MHz) δ: 173.8 (C=O, C-1'), 162.0 (C=O, C-24), 151.6 (CH, C-21), 148.6 (CH, C-22), 116.3 (C, C-20), 114.1 (CH, C-23), 75.2 (C, C-5), 74.5 (CH, C-16), 72.5 (C, C-14), 68.3 (CH, C-3), 59.6 (CH, C-15), 50.4 (CH, C-17), 45.2 (C, C-13), 42.9 (CH, C-9), 41.0 (C, C-10), 40.2 (CH_2, C-12), 36.8 (CH_2, C-4), 34.5 (CH_2, C-6), 32.3 (CH, C-8), 28.0 (CH_2, C-2), 27.5 (CH_2, C-2'), 24.9 (CH_2, C-1), 23.2 (CH_2, C-7), 21.5 (CH_2, C-11), 17.3 (CH_3, C-18), 16.9 (CH_3, C-19), 9.1 (CH_3, C-3').

Bufalin (**7**) [2]: white solid; ^{1}H-NMR ($CDCl_3$, 400 MHz) δ: 7.83 (1H, dd, *J* = 2.4, 9.7, H-22), 7.21 (1H, d, *J* = 1.9, H-21), 6.26 (1H, d, *J* = 9.8, H-23), 4.13 (1H, m, H-3), 0.93 (3H, s, H-19), 0.68 (3H, s, H-18). ^{13}C-NMR ($CDCl_3$, 100 MHz) δ: 162.8 (C=O, C-24), 148.7 (CH, C-21), 147.1 (CH, C-22), 123.0 (C, C-20), 115.5 (CH, C-23), 85.7 (C, C-14), 67.3 (CH, C-3), 51.4 (CH, C-17), 48.6 (C, C-13), 42.6 (CH, C-8), 41.1 (CH_2, C-12), 36.2 (CH, C-5), 35.9 (CH, C-9), 35.6 (C, C-10), 33.4 (CH_2, C-4), 32.9 (CH_2, C-15), 29.8 (CH_2, C-1), 28.9 (CH_2, C-16), 28.0 (CH_2, C-2), 26.7 (CH_2, C-6), 23.9 (CH_3, C-19), 21.6 (CH_2, C-11), 21.6 (CH_2, C-7), 16.7 (CH_3, C-18).

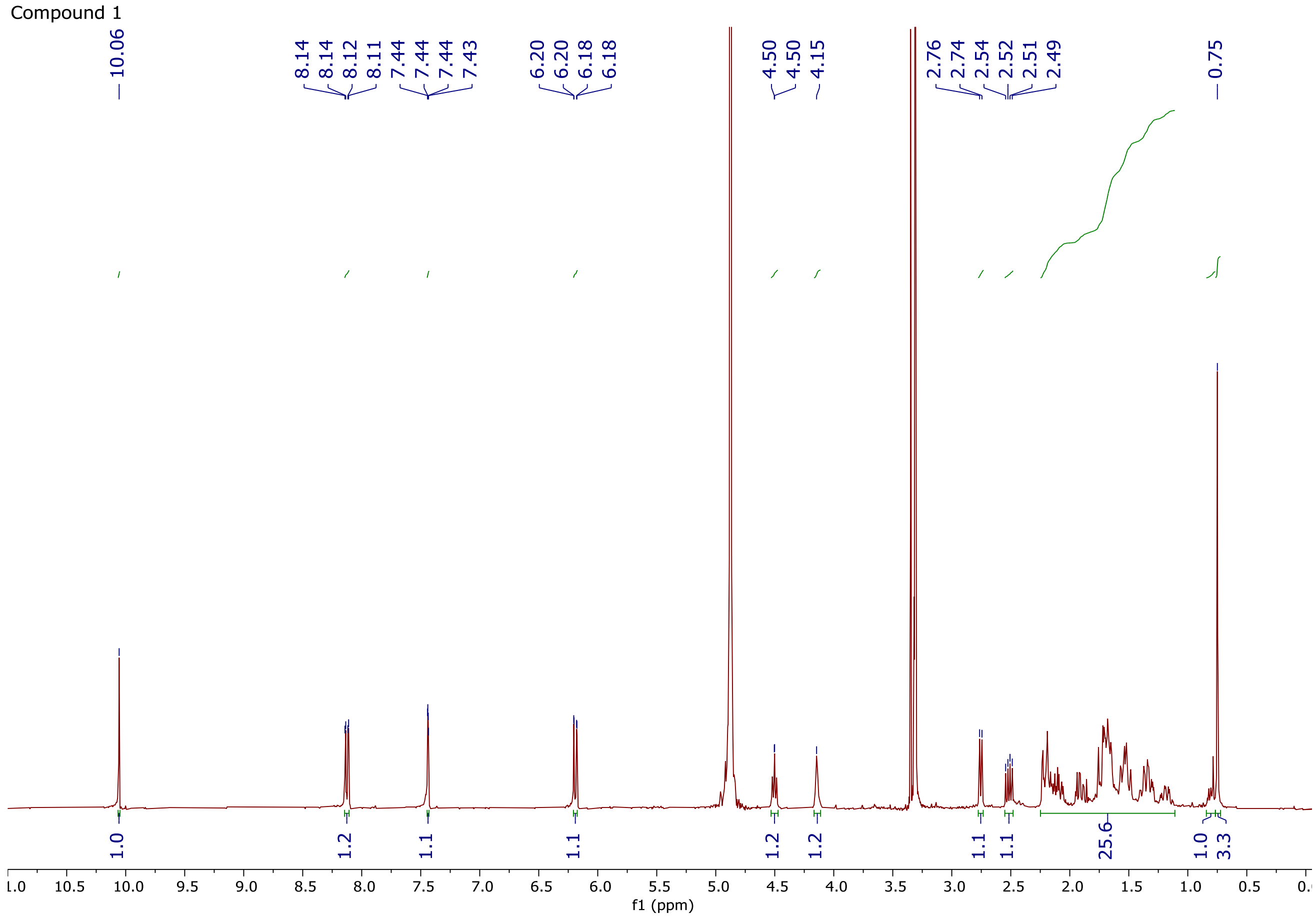

Figure S10. ^{1}H NMR spectrum (in methanol-d$_4$, 400 MHz) of compound **1** (16β-hydroxyl-hellebrigenin)

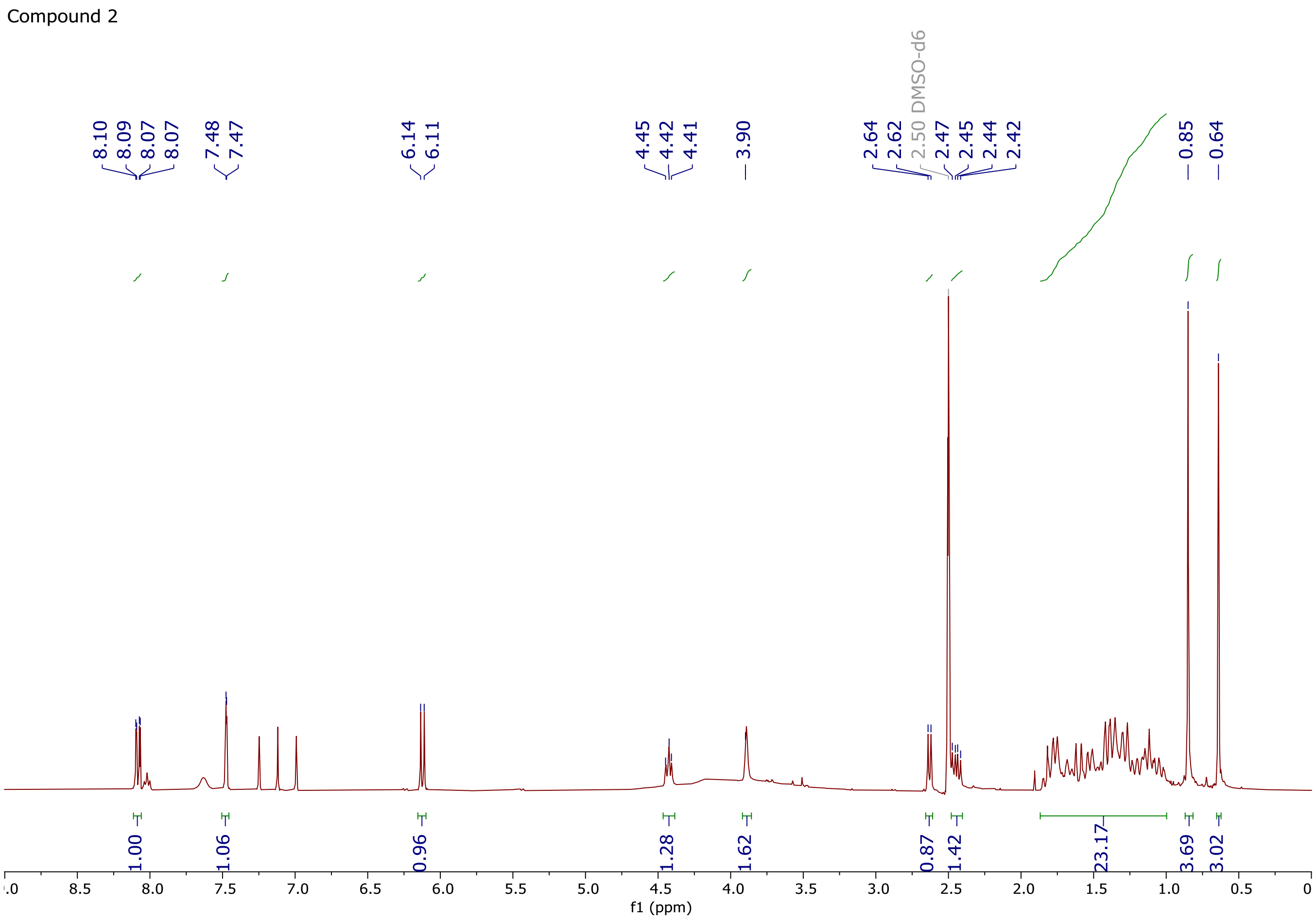

Figure S11. ^{1}H NMR spectrum (in DMSO-d, 400 MHz) of compound **2** (desacetyl-bufotalin)

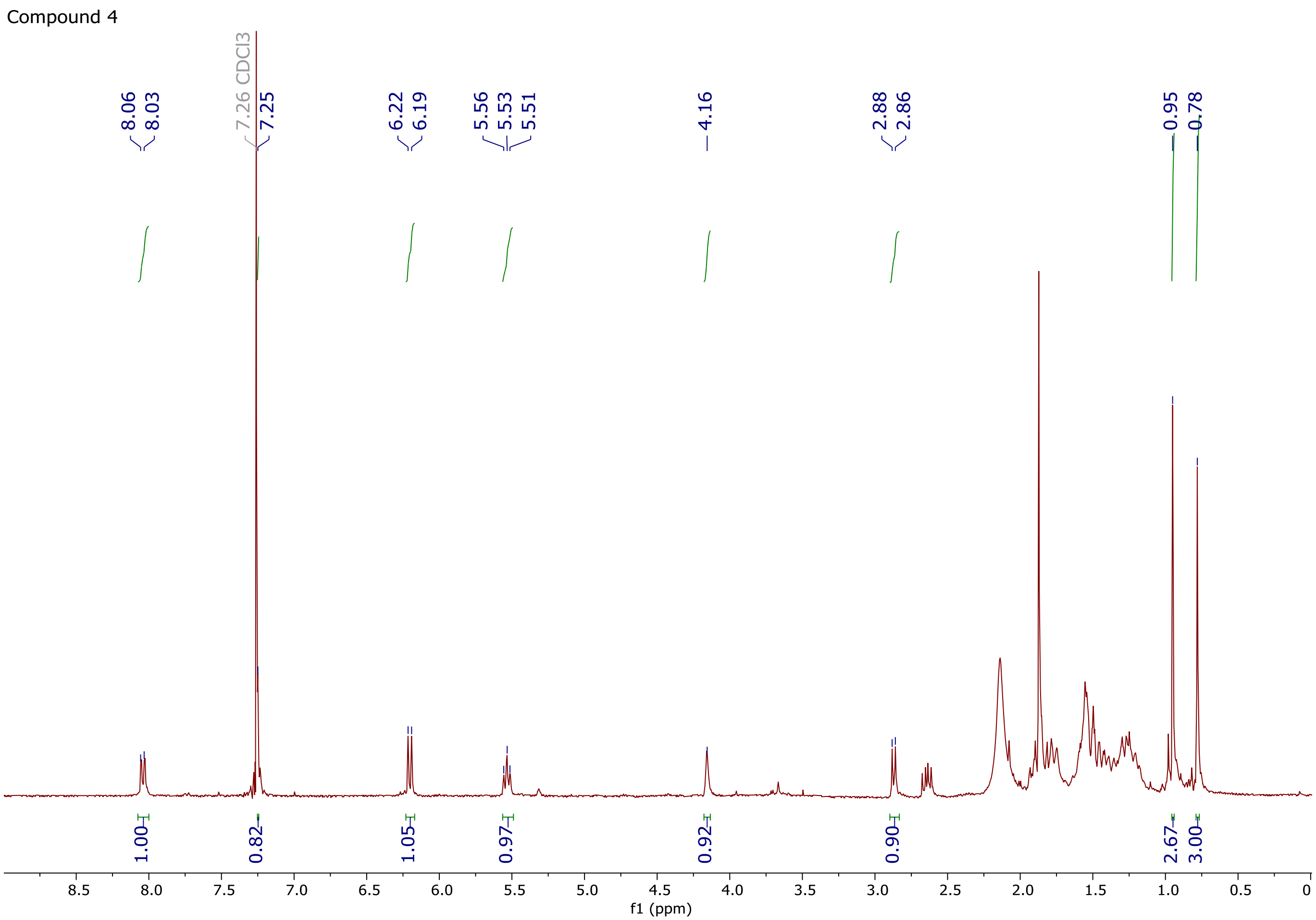

Figure S12. ^{1}H NMR spectrum of (in $CDCl_3$, 400 MHz) compound **4** (bufotalin)

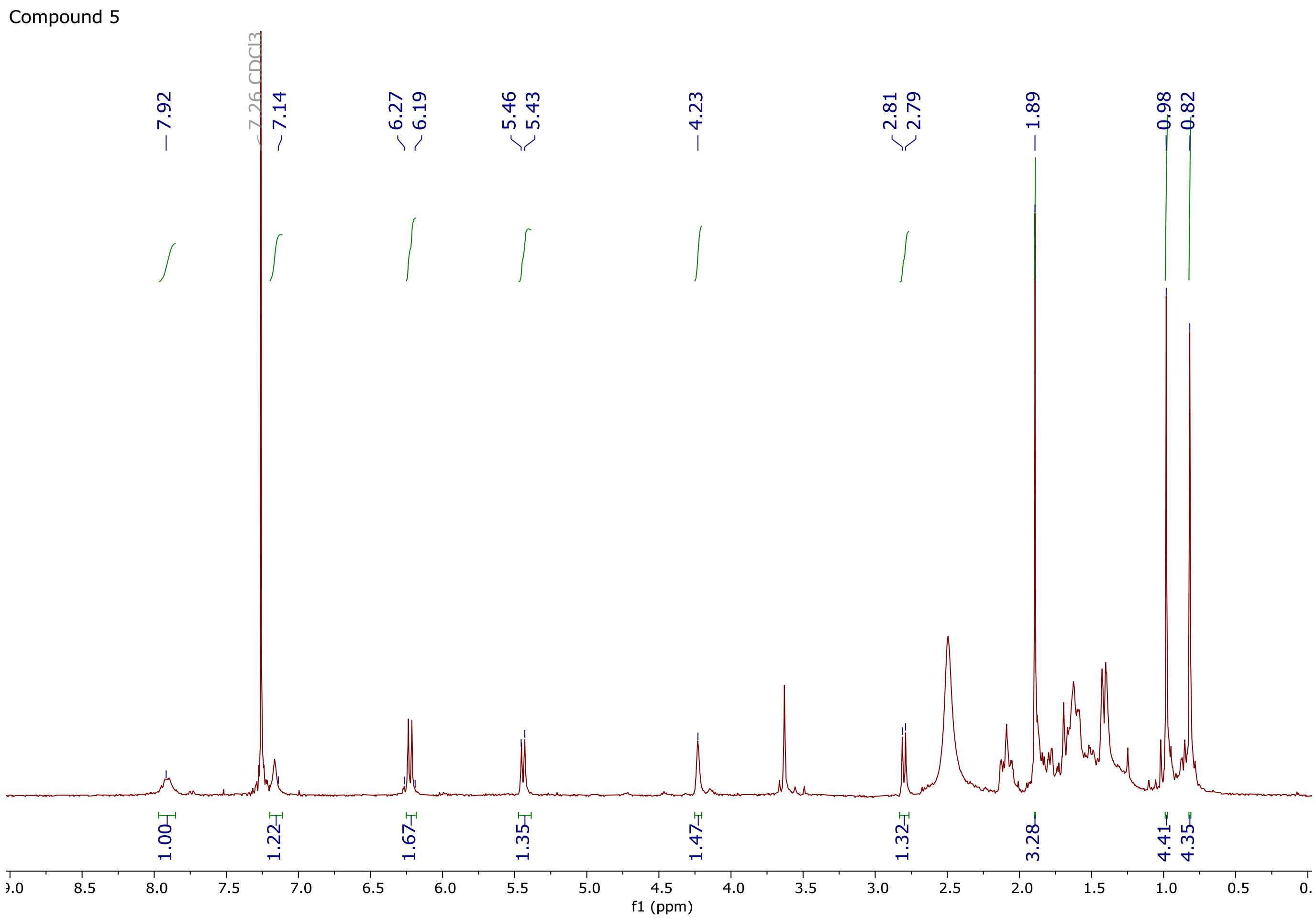

Figure S13. ^{1}H NMR spectrum (in $CDCl_3$, 400 MHz) of compound **5** (cinobufotalin)

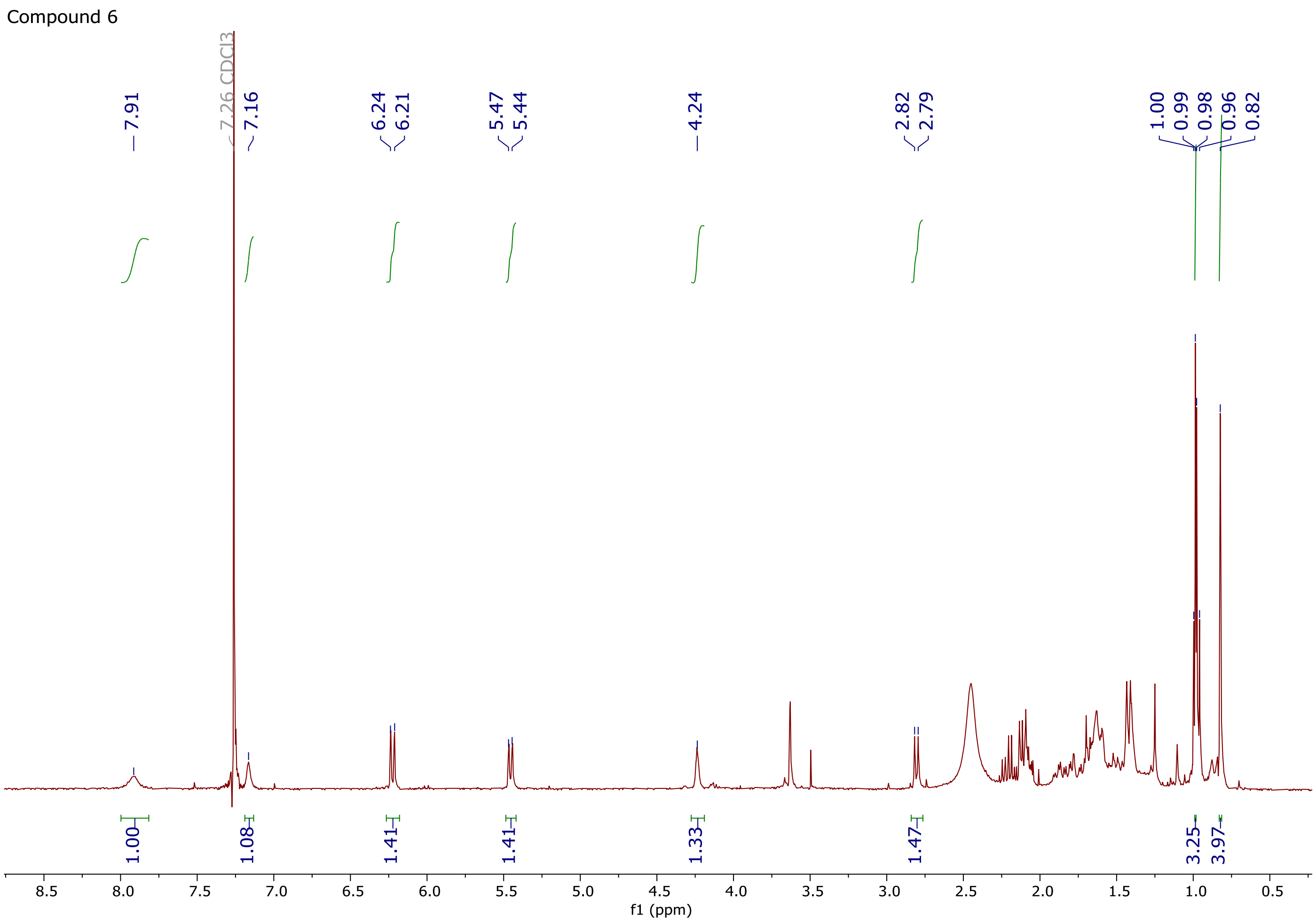

Figure S14. ^{1}H NMR spectrum (in $CDCl_3$, 400 MHz) of compound **6** (dyscinobufotalin)

Compound 7

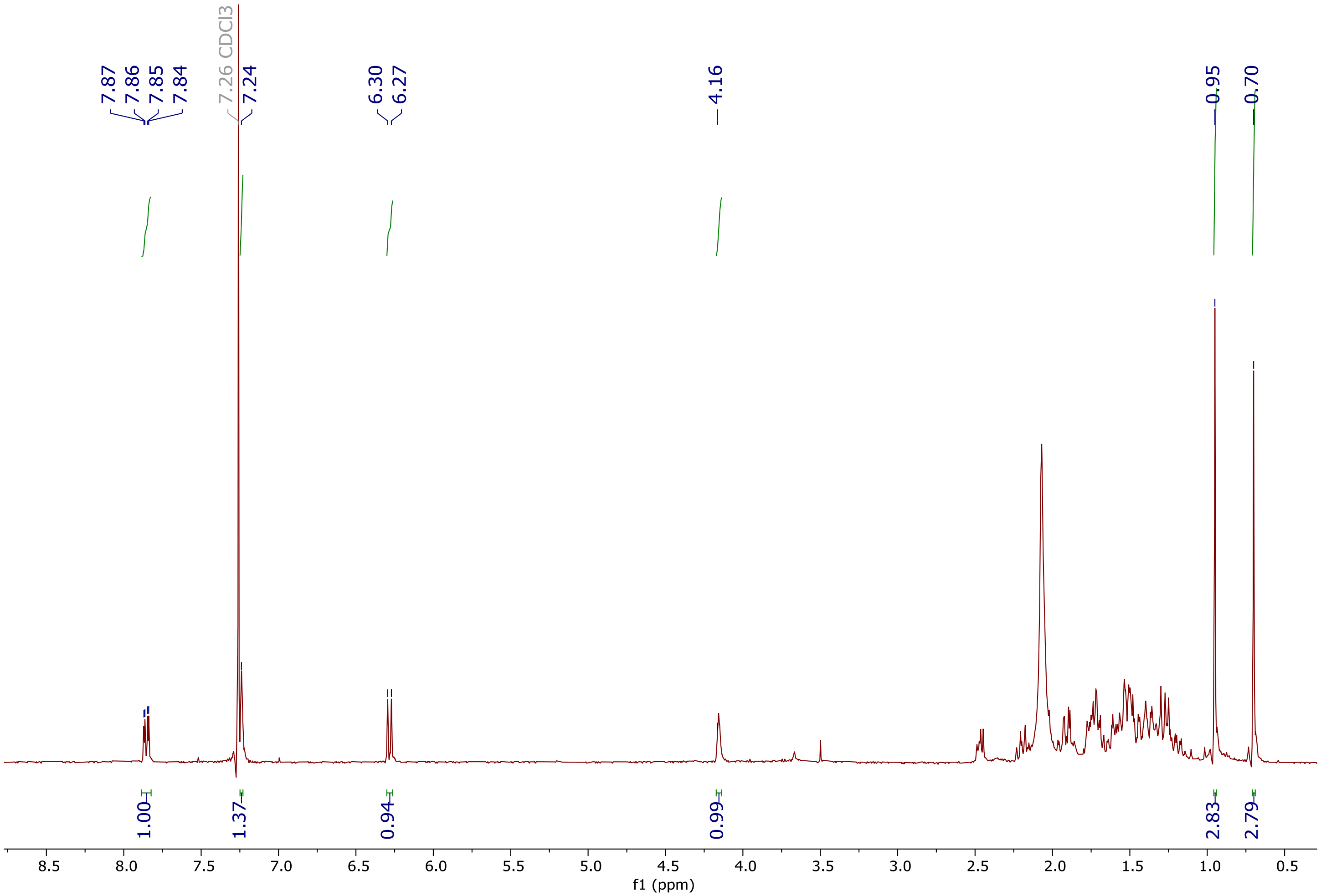

Figure S15. ^{1}H NMR spectrum (in $CDCl_3$, 400 MHz) of compound **7** (bufalin)

References

1. Krenn L., Stapf V., Kopp B. Bufadienolides from *Drimia robusta* BAK. *Sci. Pharm.* 68, 421-427 (2000).
2. Verpoorte, R.; Phax-quôc-Kinh, and Svendsen, A.B. Chemical constituents of Vietnamese toad venom, collected from *Bufo Melanostictus* Schneider. Part II. The bufadienolides. *J. Nat. Prod.* 1980, 43 (3), 347–352. DOI: 10.1021/np50009a005
3. Kamano, Y.; Nogawa, T.; Yamashita, A. and Pettit, G.R. The 1H and 13C NMR chemical shift assignments for thirteen bufadienolides isolated from the traditional Chinese drug Ch'an Su. Collect. Czechoslov. *Chem. Commun.* 2001, 66 (12), 1841–1848. DOI: 10.1135/cccc20011841
4. Xiao, J.; Zhao, X.; Zhong, W.T.; Jiao, F.R.; Wang, X.L.; Ma, L.; Duan, D.Z.; Yang, D.S. and Tang, S.Q. Bufadienolides from the venom of *Bufo gargarizans* and their enzyme inhibition activities and brine shrimp lethality. *Nat. Prod. Commun*. 2018, 13 (7), 827–830. DOI: 10.1177/1934578X1801300710
